# Cardiometabolic Risk Factors Related to Atrial Fibrillation and Metabolic Syndrome in the Pakistani Population

**DOI:** 10.3390/medicina60081190

**Published:** 2024-07-23

**Authors:** Saira Rafaqat, Saima Sharif, Shagufta Naz, Sanja Gluscevic, Filiz Mercantepe, Ana Ninic, Aleksandra Klisic

**Affiliations:** 1 Department of Zoology, Lahore College for Women University, Lahore 44444, Punjab, Pakistan; 2 Department of Neurology, Clinical Center of Montenegro, 81000 Podgorica, Montenegro; 3 Department of Endocrinology and Metabolism, Faculty of Medicine, Recep Tayyip Erdogan University, 53100 Rize, Turkey; 4 Department for Medical Biochemistry, Faculty of Pharmacy, University of Belgrade, 11158 Belgrade, Serbia; 5 Faculty of Medicine, University of Montenegro, 81000 Podgorica, Montenegro; 6 Center for Laboratory Diagnostics, Primary Health Care Center, 81000 Podgorica, Montenegro

**Keywords:** atrial fibrillation, uric acid, kidney parameters, metabolic syndrome, inflammation

## Abstract

*Background and Objectives*: This study aimed to examine the relationship between cardiometabolic risk factors and atrial fibrillation (AF) and the simultaneous presence of AF and metabolic syndrome (MetS) in the Pakistani population. *Materials and Methods*: A total of 690 subjects were enrolled (n = 230 patients with AF, n = 230 patients with AF and MetS, and n = 230 controls). The associations between cardiometabolic parameters and AF with and without MetS were analyzed by univariable and multivariable binary regression analyses. *Results*: Body mass index (BMI), fasting blood glucose (FBG), and triglycerides (TG) were independently positively correlated, but the glomerular filtration rate (GFR) and sodium were independently negatively correlated with AF. An increase in BMI, FBG, and TG levels by one unit measure increased the probability by 55.1%, 20.6%, and 1.3%, respectively, for the AF occurrence. A decrease in GFR and sodium levels increased the probability by 4.3% and 33.6%, respectively, for the AF occurrence. On the other hand, uric acid was independently negatively correlated, whereas sodium was independently positively correlated, with MetS and AF. A decrease in uric acid levels and an increase in sodium levels by 1 unit measure increased the probability for MetS and AF by 23.2% and 7.5%, respectively. *Conclusions*: Cost-effective and routinely measured parameters, i.e., BMI, FBG TG, GFR, and sodium levels, can be reliable indicators of AF, whereas serum uric acid and sodium levels are independently associated with AF and MetS in the Pakistani population. Timely recognition and the control of modifiable cardiometabolic risk factors are of great significance in the prevention of AF development.

## 1. Introduction

The prevalence of atrial fibrillation (AF) is increasing dramatically and has become a global public health concern [1,2]. It is estimated that AF affects about 33.5 million people worldwide [3], and this number is expected to increase to more than 60% by 2050 [2].

The underlying mechanisms of AF pathophysiology are not clarified yet. However, oxidative stress and inflammation are assumed to be the key triggers of this process [2,3], including the activation of the renin–angiotensin–aldosterone system, enlargement of the left atrium, impaired relaxation of the heart during its resting phase (diastolic dysfunction), and the presence of fibrous tissue in the heart muscle (myocardial fibrosis) [4], which significantly amplify the likelihood of experiencing severe consequences such as excessive bleeding, ischemic stroke, and even mortality [5].

Recent studies show that metabolic syndrome (MetS) could be a potential contributor to the triggering of AF [6,7]. MetS is a cluster of different medical conditions; it requires the presence of at least three of the following five disturbances, i.e., high fasting glucose, high blood pressure (BP), high serum triglycerides (TG), low serum high-density lipoprotein cholesterol (HDL-c), and abdominal obesity. According to the International Diabetes Federation’s results, 33.2% of Pakistan’s seemingly healthy population has MetS [8].

A nationwide population-based study of Asians recorded the association between MetS status and an increased risk of AF [9]. Likewise, similar results were confirmed by a meta-analysis that showed the association between MetS and its components with the increased risk of AF in different populations [10]. Specifically, epidemiological studies showed similar observations in samples of both Western and Asian populations, pointing out that individuals with MetS are more likely to develop AF compared to individuals without MetS [10].

Inflammation is an underlying feature of both AF and MetS. Furthermore, their simultaneous presence is linked with even higher inflammation, thus potentially including the other mechanisms that lead to AF onset and progression [6].

The simultaneous presence of these two entities remains a challenge regarding the timely recognition and the treatment of such patients. Serum biomarkers have recently been shown to be useful parameters with good diagnostic ability in AF risk stratification [2].

High uric acid is assumed to be a contributing factor to the onset/progression of AF [6]. Uric acid is an organic compound with a heterocyclic structure, weighing approximately 168 daltons. Uric acid is produced through the final stage of the metabolic breakdown of purine nucleotides by xanthine oxidase, and it is a natural component of urine, primarily eliminated from the body through the kidneys, with a smaller amount excreted through feces [11]. Hyperuricemia often occurs in patients with MetS, which is a part of its pathophysiology, although it is not a part of the criteria for diagnosing MetS [11]. 

Diabetes is also a strong independent AF risk factor, acting via different mechanisms, i.e., by inducing the dysfunction of the autonomic nervous system and via diminished insulin signaling pathways. Insulin resistance, which is the underlying feature of obesity and related disorders, is followed by an increase in C-reactive protein in the myocardium, resulting in a higher inflammation level. This inflammatory process can aggravate diastolic dysfunction by promoting atrial dilation and myocardial fibrosis [10].

Obesity-related chronic low-grade inflammation and the concomitant insulin resistance also affect thyroid function. Thyroid dysfunction is also one of the mechanisms that play a role in the pathogenesis of AF [12]. Thyroid dysfunction initiates the occurrence of atrial fibrosis, thus increasing the risk for AF [12].

Animal studies show that hypertension, as a component of MetS, can promptly promote the hypertrophy of the left atrium, inflammation, and fibrosis. Electrophysiological changes, such as changes in Ca^2+^ current density and shortened atrial wavelength, may also occur and can all contribute to an increased AF risk [10]. It is also important to note that regardless of hypertension, MetS predisposes to the development of left ventricular myocardium hypertrophy and left ventricular diastolic dysfunction, which is also linked with an increase in the volume of the left atrium, thus promoting the development of AF [12].

However, the knowledge gap between the pathophysiological mechanisms related to AF and MetS needs to be further explored. Timely recognition and the control of modifiable cardiometabolic risk factors are of great significance in the prevention of AF development [13]. Notably, there is a lack of data regarding the utility of the measurement of serum uric acid levels, specifically in individuals with AF and MetS in the Pakistani population. Therefore, the study aimed to examine the relationship between cardiometabolic risk factors and AF and the simultaneous presence of AF and MetS in the Pakistani population.

## 2. Material and Methods

This case–control study was conducted in the Department of Zoology, Lahore College for Women University, Lahore. The study employed a non-probability purposive sampling strategy.

The subjects were recruited from the Punjab Institute of Cardiology, Lahore, Pakistan, from July 2021 to June 2022. The subjects were enrolled after their informed consent. The study was approved by the institutional ethical review committee (ref.no: RTPGME-Research-179). All the patients provided their consent to participate in the study.

The inclusion and exclusion criteria are described in detail in our previous study [7].

The patients who were older than 18 years, without NCEP ATP III criteria for MetS, and with a previous diagnosis of coronary artery disease, transient ischemic attack, myocardial infarction, stroke, prior coronary artery bypass graft surgery, percutaneous coronary intervention, or systemic embolism were enrolled in the AF without MetS group.

During the assignment of patients with MetS, we specifically focused on those with AF with hypertension, diabetes, or taking medications for these two disorders and on waist circumference > 35 inches for women and > 40 inches for men.

However, pregnant women and individuals who planned to undergo surgery to treat AF or pulmonary vein ablation were excluded from the study.

Healthy individuals without MetS criteria and without endocrine disorders, cardiovascular disease, kidney disease, or autoimmune or malignant diseases, and those who voluntarily accepted to participate were included in the control group.

In brief, all the participants underwent anthropometric measurements (i.e., body weight, height, and waist circumference; body mass index (BMI) and waist to height ratio (WHR) were calculated), blood pressure (BP) measurements (with a sphygmomanometer and by a medically trained staff), electrocardiography (ECG), and blood sampling. 

Each participant filled in the questionnaire regarding the demographic data, history of the diseases, medication use, smoking, alcohol consumption, and physical activity.

**Figure 1:**
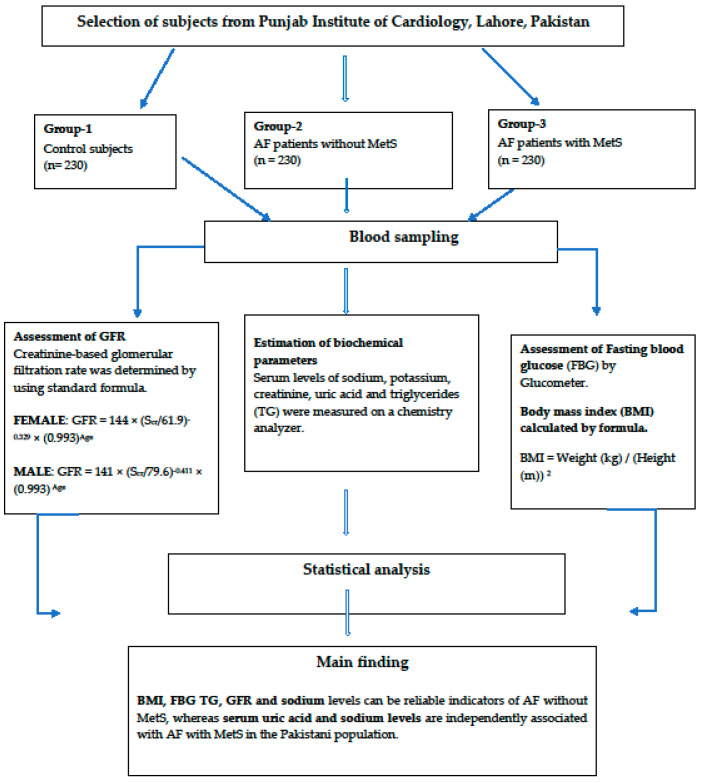
Flowchart of the selected participants.

A flowchart of the selected participants is shown in Figure 1.

### 2.1. Electrocardiography

The study subjects with AF were selected based on their diagnosis by physicians at the Punjab Institute of Cardiology using ECG, which showed the absence of P waves, disorganized electrical activity, and irregular R-R intervals due to irregular conduction of impulses to the ventricles.

### 2.2. Diagnosis of Metabolic Syndrome

The National Cholesterol Education Program Adult Treatment Panel III (NCEP ATP III) criteria were used to diagnose MetS [7]. If the participants met any 3 or more of the following criteria, they were assumed to have MetS, i.e., waist circumference for women > 35 inches and for men > 40 inches; TG ≥ 150 mg/dL; HDL-c for women < 50 mg/dL and for men < 40 mg/dL; fasting blood glucose (FBG) ≥ 100 mg/dL and hypertension [systolic BP (SBP) ≥ 130 mmHg or diastolic BP (DBP) ≥ 85 mmHg].

### 2.3. Collection of Blood Sample

The blood samples were collected and processed for serum separation. The serum separation tube contained a separating gel and was centrifuged at 3000 rpm for 15 min after 30 min to obtain serum samples. The samples were stored at a temperature of −80 °C until further analysis.

### 2.4. Estimation of Biochemical Parameters

The FBG, serum levels of sodium, potassium, creatinine, uric acid, and TG of the studied subjects were measured using a chemistry analyzer (ERBA Chemistry Analyzer, Model # CHEM-7, Serial # 9047, ERBA Diagnostics, Mannheim GmbH, Germany) in the research laboratory of Lahore College for Women University with commercially available kits. 

### 2.5. Assessment of GFR

The creatinine-based glomerular filtration rate [14] was determined using the standard formula.

For women: GFR = 144 × (S_cr_/61.9)^−0.329^ × (0.993)^Age^

For men: GFR = 141 × (S_cr_/79.6)^−0.411^ × (0.993)^Age^

### 2.6. Statistical Analysis 

The study’s sample size was determined using the Rao software, taking into account the disease’s prevalence and a 5% margin of error. A total of 690 participants were included in the study, which comprised three groups: a control group of 230 healthy individuals with no history of AF, diabetes, or hypertension; a group of 230 subjects with AF, but without MetS; and a group of 230 patients with both AF and MetS, based on the specified inclusion and exclusion criteria.

Statistical analyses were conducted using the Statistical Package for Social Sciences version 21.0 (SPSS Inc., Chicago, IL, USA). The data distribution was analyzed by Kolmogorov–Smirnov test. The intergroup differences in anthropometric, clinical, and biochemical continuous variables were evaluated using the Kruskal–Wallis test for 3 groups and Mann–Whitney U test for 2 groups. The results were reported as median values (interquartile range). The categorical variables were compared between the groups using the chi-squared test for contingency tables, and the results were reported as absolute and relative frequencies. The associations between the study data (independent variables) and AF, and MetS in AF (dependent variables) were analyzed by univariable and multivariable binary regression analyses. The results were reported as the odds ratio (OR) and 95% confidence interval. Nagelkerke R^2^ values demonstrated the explained variations in AF and MetS in the AF presence. The statistical tests were two-sided, and *p* values less than 0.05 were considered as significant.

## 3. Results

Table 1 presents the anthropometric and clinical data of the examined population. The groups were homogenous regarding sex distribution and years of age. The patients with AF and MetS had a greater history of heart attack than the patients with AF only. The participants of both patient groups had higher SBP compared to the controls. Drug usage was evident among the patients with AF without and with MetS compared to the controls. Although similar drug usage was shown in all the patients, those with AF and MetS used antihypertensives less frequently. The examinees with AF had the highest BMIs, and the controls had the lowest BMIs compared to the other groups. 

The serum FBG and TG levels were the higher in the patients with AF and MetS than in two other groups (Table 2). The levels of these biochemical markers were also higher in the patients with AF than in the controls. The serum uric acid levels were also significantly different between all the tested groups, with the highest levels in the patients with AF without MetS and the lowest in the patients with AF with MetS. Conversely, higher concentrations of sodium and potassium were shown in the controls than in the other groups. The sodium concentration was the lowest in the patients with AF, but the potassium concentration was the lowest in the patients with AF and MetS. The creatinine levels and GFR were also significantly different between the groups. The creatinine levels were higher, but GFR was lower in both patient groups than in the controls.

The associations of the anthropometric, clinical, and biochemical data with AF without MetS and AF with MetS were assessed by univariable and multivariable binary regression analyses (Table 3, Table 4 and Table 5). 

The results showed that BMI, SBP, FBG, TG, uric acid, creatinine, and potassium were positively associated with AF without MetS (Table 3). An increase in BMI, SBP, FBG, TG, uric acid, creatinine, and potassium by one unit measure increased the probability by 36%, 5,6%, 13.8%, 1.1%, 23.6%, 75 times, and 13.3%, respectively, for the AF occurrence. The DBP, GFR, and sodium levels were negatively associated with AF. A decrease in the DBP, GFR, and sodium levels by 1 unit measure increased the probability by 2%, 3.6%, and 25.6%, respectively, for the AF occurrence.

The univariable binary regression analysis for testing the associations of clinical and biochemical markers with AF with MetS included those markers that were not considered for MetS diagnosis (Table 4). The uric acid and potassium levels were negatively associated and the sodium levels was positively associated with AF with MetS. A decrease in uric acid and potassium levels and an increase in sodium levels by 1 unit measure increased the probability for AF with MetS by 23.2%, 2.5%, and 6%, respectively. 

The models in the multivariable binary regression analyses for the independent associations of tested markers with AF without MetS and AF with MetS were different (Table 5). Model 1 included all the markers that showed significant ORs in the univariable analysis for the association with AF without MetS. Also, this model was able to explain a total of 90.1% in AF variation. BMI, FBG, and TG demonstrated the independent positive associations, but GFR and sodium demonstrated the independent negative associations with AF without MetS. An increase in BMI, FBG, and TG levels by one unit measure increased the probability by 55.1%, 20.6%, and 1.3%, respectively, for the AF occurrence. A decrease in GFR and sodium levels increased the probability by 4.3% and 33.6%, respectively, for the AF occurrence. 

When adjusted for antihypertensive therapy in Model 2, uric acid demonstrated the independent negative association and sodium demonstrated the independent positive association with AF with MetS, whereas potassium lost its independent association with AF with MetS (Table 5). A decrease in uric acid levels and an increase in sodium levels by 1 unit measure increased the probability for AF with MetS by 23.2% and 7.5%, respectively. The coefficient of determination (R^2^) for Model 2 was 0.185. Using this model, the variation in AF with MetS can be explained by 18.5%.

## 4. Discussion

The purpose of this research was to assess the role of cardiometabolic risk factors in AF subjects with and without MetS in the Pakistani population. The main findings of the current study show that BMI, FBG, and TG were independently positively correlated, but GFR and sodium were independently negatively correlated with AF without MetS. On the other hand, uric acid was independently negatively correlated, whereas sodium was independently positively correlated with AF with MetS. To the best of our knowledge, no previous study examined the role of uric acid in AF with and without MetS.

Over 650 million individuals worldwide are affected by the obesity pandemic [15]. It is one of the best examples of modifiable risk factors linked to cardiometabolic diseases, including AF and MetS, which all contribute to the high rates of morbidity and mortality among individuals with obesity. Obesity acts through several pathophysiological mechanisms, including elevated plasma levels of free fatty acids and pro-inflammatory cytokines and adipokines, intracellular non-adipose tissue lipids (like liposomes), and ectopic adipose tissue depots (especially the visceral compartment), which can cause oxidative stress, systemic inflammation, insulin resistance, and sympathetic nervous system hyperactivity [15,16,17]. Insulin resistance, as the underlying feature of obesity, MetS, and diabetes, is followed by an increase in inflammation level which enhances the atrial dilation and myocardial fibrosis, diastolic dysfunction, and consequent AF [10].

The increase in adipose tissue and body weight also influences thyroid function. Hyperleptinemia, which occurs in obesity-related metabolic disturbances, favors the synthesis of the thyrotropin-releasing hormone via the promotion of the synthesis of the α-melanocyte-stimulating hormone and the activation of the STAT3 transcription factor [12]. On the other hand, thyroid dysfunction (primarily hyperthyroidism) is related to an increased risk of AF. Thyroid hormones influence cardiac functional and structural parameters and electrical remodeling, as shown by animal models and experimental research at the cellular level [18]. The atrial myocardium is very susceptible to thyroid hormones, and thyroid dysfunction initiates the occurrence of atrial fibrosis, thus increasing the risk for AF [18].

The results of our study show that TG and FBG have an independent positive association with AF without MetS. Similarly, having high FBG levels and undiagnosed diabetes pose the risks for the eventual development of both AF and heart failure. Ref. [13] However, the relationship between blood lipid profiles and the risk of AF is still a topic of debate. Contrary to our study, no significant association was found between TG levels and the occurrence of AF [19].

Hyperlipidemia is assumed to be a potential contributor to AF onset via the creation of a milieu of increased inflammation and oxidative stress [6,20]. A previous study showed that patients with AF exhibited higher levels of inflammation parameters, as well as 1.6-fold higher plasma TG compared to the patients without AF [21]. Moreover, an association between postprandial very low-density lipoprotein (VLDL), composed of TG, and atrial remodeling in subjects with MetS was confirmed, suggesting that dyslipidemia may affect electrical and structural modifications in the cardiac chambers [22]. However, future investigations are needed in this area.

In the present study, serum uric acid was independently negatively associated with AF with MetS. Conversely, Zhong et al. [23] found that increased levels of uric acid were linked to AF, although the association was particularly significant among women. However, the latter study did not evaluate AF and MetS simultaneously. Additionally, the possible discrepancies between our results and the latter study might in part be attributed to the different study populations, as well as the effect of medication use. Importantly, the role of serum uric acid in cardiometabolic diseases is still controversial given the fact that uric acid exerts dual effects [24]. Specifically, serum uric acid exerts antioxidant activity by scavenging reactive oxygen species, thus showing beneficial properties and preventing atherosclerosis [24]. On the other hand, previous studies have shown that the elevation of uric acid levels may potentially contribute to the development of other factors associated with AF and actively participate in the pathological processes underlying AF [23]. Serum uric acid is suggested to be a marker for cardiovascular risk and is linked to oxidative stress and inflammation, presuming that serum uric acid could potentially be a novel risk factor for the development of AF [25]. Furthermore, uric acid can induce oxidative stress by diminishing nitric oxide production in arterial endothelial cells and impeding vasodilation. This process is thought to play a role in the pathogenesis of AF [26,27].

Our results showed that serum creatinine levels were higher, but GFR levels were lower in both the AF without MetS and the AF with MetS groups compared to the control group. According to Auer et al., patients with postoperative AF had lower baseline creatinine clearance than patients without AF [28]. GFR is an independent negative linked with AF patients. Another study explained that both eGFR and proteinuria appeared to be associated with the persistent form of AF, but their contribution to the pathogenesis does not appear to exceed the atrial stretch and remodeling represented by the left ventricular ejection fraction and left atrial dimension [29].

The results of the current study also showed higher serum levels of sodium in the controls than in the other groups, with the lowest being in the patients with AF without MetS. Moreover, the serum sodium levels were independently positively correlated with those with AF suffering from MetS, whereas they were negatively correlated with AF without MetS. Similarly, Can et al. [30] discovered that individuals with AF had significantly lower serum sodium levels compared to the control group. The decreased serum sodium levels could contribute to the development of AF by impeding the inhibitory function of the AV node on accessory pathways. It is plausible that serum sodium levels might influence the classification of AF types. Episodes of paroxysmal AF could potentially be triggered by sudden reductions in serum sodium levels. In other words, the lower serum sodium levels observed in patients with persistent AF, which tend to rise due to compensatory mechanisms but remain below the levels of the control group, could be a causal factor for AF by obstructing the phase depolarization in the AV node. Additionally, it was revealed that individuals with paroxysmal AF exhibited lower serum sodium levels than those with permanent AF [30].

Our results showed higher serum levels of potassium in controls than in the other examined groups, with the lowest being in patients with AF and MetS. Serum potassium has been linked to ventricular arrhythmias and cardiac arrest, but the association with AF remains largely unexplored. A previous study indicated that lower levels of serum potassium were connected to an increased risk of developing AF [31]. However, in our current study we did not confirm the independent associations between serum potassium levels and AF with and without MetS, respectively.

The limitations of this study deserve to be mentioned. Since the study is cross-sectional, we cannot draw a conclusion regarding the causality between the examined cardiometabolic risk factors and AF with and without MetS. The heterogeneity of the population of patients with MetS is another limitation, and future studies that analyzed the differences between individuals with three, four, or five components of MetS could be beneficial. The non-probability purposive sampling strategy may have introduced selection bias, thus potentially affecting the representativeness of the sample. Also, it could be a worthy aim to measure the biomarkers of oxidative stress and inflammation in order to gain deeper insight into the pathophysiological mechanisms underlying AF and MetS. Therefore, future studies with a longitudinal design which offer more robust evidence for these associations and that include oxidative stress and inflammatory biomarkers are necessary to more deeply investigate this issue. The follow-up data of the study could assess how these risk factors might change over time or predict future AF or MetS development. Importantly, while the study controls for several variables, there may still be confounding factors influencing the results that are unaccounted for. A more comprehensive analysis, including additional potential confounders, would strengthen the findings. Moreover, similar studies that included different populations in different regions would enhance the applicability of the results. Although the current study did not differentiate between different types of AF (e.g., paroxysmal, persistent, or permanent), which could have different relationships with the measured risk factors, we suggest that future studies with these kinds of design are needed to enable deeper insight into the relationship between the cardiometabolic risk factors related to AF and MetS.

## 5. Conclusions

Cost-effective and routinely measured parameters, i.e., BMI, FBG TG, GFR, and sodium levels can be reliable indicators of AF without MetS, whereas serum uric acid and sodium levels are independently associated with AF with MetS in the Pakistani population. Hence, clinicians should pay attention to these modifiable risk factors, since timely recognition and control of these risk factors are of great significance in the prevention of the AF development. New studies with a larger sample size and with longitudinal design are needed to explore the causal association between the examined parameters and AF with and without MetS.

## Figures and Tables

**Table 1:** Anthropometric and clinical characteristics of studied groups.

Variables	Controls (n = 230)	AF without MetS (n = 230)	AF with MetS (n = 230)	*p*
Male, N (%) ^‡^	108 (47)	120 (52)	106 (70)	0.369
Age (years)	60 (50–70)	60 (50–70)	60 (50–70)	0.607
BMI (kg/m^2^)	23.3 (21.6–24.1)	28.5 (22.8–34.5) ^a^*	23.0 (19.6–34.5) ^a†,b#^	<0.001
WHR	0.80 (0.76–0.86)	0.80 (0.76–0.86)	0.68 (0.91–0.97)	0.474
SBP (mmHg)	110 (110–120)	120 (110–150) ^a^*	120 (100–160) ^a^*	<0.001
DBP (mmHg)	90 (80–90)	90 (70–90)	90 (90–100)	0.704
Kidney disease, N (%) ^‡^	0 (0)	16 (7) ^a^*	24 (10) ^a^*	<0.001
Valve problems, N (%) ^‡^	0 (0)	15 (6.5) ^a^*	18 (7.7) ^a^*	<0.001
Drug usage, N (%) ^‡^				<0.001
Antihypertensive	0 (0)	65 (28) ^a^*	45 (20) ^a^*^,b†^	
Antiplatelet	0 (0)	30 (13) ^a^*	32 (14) ^a^*	
Antiarrythmic	0 (0)	77 (33) ^a^*	93 (40) ^a^*	
Anticoagulants	0 (0)	51 (53) ^a^*	45 (20) ^a^*	
Opioid Analgesic	0 (0)	7 (3) ^a^*	15 (6) ^a^*	

Data were compared by Kruskal–Wallis and Mann–Whitney tests and given as median (interquartile range). ^‡^ Data were compared by chi-square test and given as absolute and relative frequencies. ^a^—significantly different from control group; ^b^—significantly different from AF without MetS. * *p* < 0.001; ^#^ *p* < 0.01; ^†^ *p* < 0.05.

**Table 2:** Biochemical markers of the study groups.

Variables	Controls (n = 230)	AF without MetS (n = 230)	AF with MetS (n = 230)	*p*
FBG (mg/dL)	89 (84–90)	115 (97–126) ^a^*	127 (115–150) ^a^*^,b^*	<0.001
TG (mg/dL)	57.3 (41.0–131.0)	155.1 (92.8–170.4) ^a^*	160.0 (150.0–178.1) ^a^*^,b#^	<0.001
Uric acid (mg/dL)	6.18 (4.74–7.63)	6.94 (4.87–8.73) ^a^*	5.11 (4.17–7.23) ^a#,b^*	<0.001
Creatinine (mg/dL)	0.6 (0.5–0.7)	0.9 (0.7–1.4) ^a^*	0.9 (0.7–1.5) ^a^*	<0.001
GFR (mL/min/1.73 m^2^)	113 (102–122)	93 (58–112) ^a^*	98 (60–117) ^a^*	<0.001
Sodium (mmol/L)	139 (136–140)	135 (132–137) ^a^*	137 (132–139) ^a^*^,b#^	<0.001
Potassium (mmol/L)	4.2 (3.9–4.6)	4.1 (3.8–5.1)	4.1 (3.6–4.2) ^a^*^,b^*	0.778

^a^—significantly different from control group; ^b^—significantly different from AF without MetS; * *p* < 0.001; ^#^ *p*< 0.01.

**Table 3:** Associations of clinical and laboratory markers with AF using univariable binary regression analysis.

	OR (95% CI)	*p*	Nagelkerke *R*^2^
**Dependent Variable: 0—Controls, 1—AF without MS**			
Age (years)	1.004 (0.988–1.021)	0.608	0.001
BMI (kg/m^2^)	1.366 (1.287–1.450)	<0.001	0.423
WHR	0.333 (0.009–12.270)	0.550	0.001
SBP (mmHg)	1.056 (1.042–1.071)	<0.001	0.213
DBP (mmHg)	0.980 (0.963–0.998)	0.030	0.014
FBG (mg/dL)	1.138 (1.112–1.165)	<0.001	0.588
TG (mg/dL)	1.011 (1.008–1.014)	<0.001	0.168
Uric acid (mg/dL)	1.236 (1.145–1.334)	<0.001	0.096
Creatinine (mg/dL)	75.000 (25.838–217.698)	<0.001	0.366
GFR (mL/min/1.73 m^2^)	0.964 (0.956–0.972)	<0.001	0.270
Sodium (mmol/L)	0.744 (0.697–0.794)	<0.001	0.334
Potassium (mmol/L)	1.133 (1.014–1.265)	0.027	0.093

**Table 4:** Associations of clinical and laboratory markers with MetS in patients with AF.

	OR (95% CI)	*p*	Nagelkerke *R*^2^
**Dependent variable: 0—AF without MetS, 1—AF with MetS**			
Age (years)	1.000 (0.984–1.016)	1.000	0.001
Uric acid (mg/dL)	0.768 (0.710–0.832)	<0.001	0.146
Creatinine (mg/dL)	0.972 (0.852–1.108)	0.669	0.001
GFR (mL/min/1.73 m^2^)	1.004 (0.999–1.010)	0.101	0.008
Sodium (mmol/L)	1.060 (1.016–1.106)	0.007	0.021
Potassium (mmol/L)	0.975 (0.956–0.994)	0.009	0.021

**Table 5:** Multivariable binary regression analysis for the associations of clinical and laboratory markers with AF without MetS and AF with MetS.

	OR (95% CI)	*p*	Nagelkerke *R*^2^
**Dependent variable: 0** **—** **Control group, 1** **—** **AF without MetS** **Model 1**			
BMI (kg/m^2^)	1.551 (1.346–1.787)	<0.001	0.901
FBG (mg/dL)	1.206 (1.143–1.273)	<0.001	
TG (mg/dL)	1.013 (1.007–1.020)	<0.001	
Uric acid (mg/dL)	1.159 (0.952–1.412)	0.142	
GFR (mL/min/1.73 m^2^)	0.957 (0.938–0.976)	<0.001	
Sodium (mmol/L)	0.664 (0.553–0.797)	<0.001	
Potassium (mmol/L)	1.076 (0.939–1.232)	0.294	
**Dependent variable: 0** **—** **AF without MetS, 1** **—** **AF with MetS** **Model 2**			
Uric acid (mg/dL)	0.768 (0.708–0.833)	<0.001	0.185
Sodium (mmol/L)	1.075 (1.028–1.124)	0.002	
Potassium (mmol/L)	0.989 (0.968–1.010)	0.293	

Model 2 was adjusted for antihypertensive therapy (categorical variable).

## Data Availability

The data will be available upon reasonable request (contact person: aleksandranklisic@gmail.com).
